# Comparison Between SGLT2 Inhibitors and Lactation: Implications for Cardiometabolic Health in Parous Women

**DOI:** 10.1089/met.2024.0182

**Published:** 2025-03-06

**Authors:** Maria A. Ramos-Roman

**Affiliations:** Department of Internal Medicine, Division of Endocrinology, University of Texas Southwestern Medical Center, Dallas, Texas, USA.

**Keywords:** breastfeeding, cardiovascular, GLUT1, lactation, postpartum, SGLT2

## Abstract

Sodium-glucose cotransporter-2 (SGLT2) inhibition and lactation result in the excretion of large amounts of glucose in urine or milk and are associated with a lower risk of cardiovascular events. The respective mechanisms behind this association with cardiovascular protection are not clear. This review compares the contribution of noninsulin-mediated glucose transport during pharmacologic inhibition of SGLT2 with noninsulin-mediated glucose transport during lactation in terms of the implications for the cardiometabolic health of parous women. The search topics used to obtain information on SGLT2 inhibitors included mechanisms of action, atherosclerosis, and heart failure. The search topics used to obtain information on lactation included cardiovascular health and milk composition. Subsequent reference searches of retrieved articles were also used. Active treatment with SGLT2 inhibitors affects glucose and sodium transport in the kidneys and predominantly protects against hospitalization for heart failure soon after the onset of therapy. Active lactation stimulates glucose transport into the mammary gland and improves subclinical and clinical atherosclerotic vascular disease years after delivery. Both SGLT2 inhibitors and lactation have effects on a variety of glucose transporters. Several mechanisms have been proposed to explain the cardiometabolic benefits of SGLT2 inhibition and lactation. Learning from the similarities and differences between both processes will advance our understanding of cardiometabolic health for all people.

## Introduction

Excretion of glucose in body fluids like urine and milk reduces blood glucose concentrations and induces systemic effects that alter metabolism in distant organs. Increased glucose excretion can be achieved by pharmacologic or physiological means while the stimulus lasts. This stimulus can be a drug like a sodium-glucose cotransporter-2 (SGLT2) inhibitor or a physiological state like lactation. In view of the undisputed benefits of SGLT2 inhibitors on cardiovascular health and the consistent observations that lactation decreases maternal cardiovascular risk, this review compares SGLT2 inhibition and lactation as strategies that promote multiorgan protection beyond their glucose-lowering effects.

Slowing down the atherosclerosis process would likely require years of sustained improvement in risk factors for atherosclerotic cardiovascular disease (ASCVD).^1^ SGLT2 inhibitors significantly reduce hospitalization for heart failure, atherosclerotic events, cardiovascular, and total mortality as well as progression of chronic kidney disease.^2,3^ However, the reductions in adverse cardiovascular outcomes and hospitalizations for heart failure observed in clinical trials had an earlier onset than anticipated by an effect in reducing atherosclerosis. In comparison, the lifetime history of lactation combines relatively small but sustained improvements in cardiometabolic risk factors with fewer cardiovascular events.^4^

## Glucose Transport Mediated by SGLT2: Metabolic Effects of SGLT2 Inhibition

SGLT2 is a glucose sensor that transports glucose and sodium from the tubular lumen into the proximal tubule cells of the kidney independently of insulin.^5^ SGLT2 inhibitors block the SGLT2 transporter resulting in glucosuria of 50–100 grams per day depending on blood glucose concentration and drug dose.^6,7^ This amount of glucosuria decreases both glucotoxicity and the flux of glucose through the hexosamine biosynthesis pathway, improving both insulin secretion during hyperglycemic clamps and insulin sensitivity during hyperinsulinemic-euglycemic clamps.^8,9^ SGLT2 inhibitors reduce fasting glucose and insulin concentrations, characteristics that are associated with health and longevity in other contexts.^10^ SGLT2 inhibitors also increase endogenous glucose production (EGP) in the fasting state and promote lipid oxidation.^11^ This drug class may reduce body weight, visceral fat, lipotoxicity, and hepatic steatosis.^2,12^ These pharmacologic effects have a rapid onset and only persist during active treatment. SGLT2 inhibitors induce a fasting-like state characterized by increased nutrient deprivation signaling and autophagic flux, and a decrease in nutrient surplus signaling, apoptosis, and senescence.^13^ The heart does not express SGLT2 transporters.^14^

## SGLT2 Inhibitors: Mechanisms of Protection

Multiple mechanisms have been proposed to explain the organ-protective effects of SGLT2 inhibitors. These mechanisms include the impact of SGLT2 inhibitors on metabolism and hemodynamics.^15^ Specific examples are inducing a fasting-like state with shifts in energy metabolism from glucose to fat oxidation, modification of cellular life history programs, increase in hematocrit, natriuresis, decrease in blood pressure and inflammation, effects on plaque burden, size, and stability, and nonatherosclerotic mechanisms.^2,16–20^ Aggregated clinical trials data suggest a potential benefit of SGLT2 inhibitors on myocardial infarction beyond the consistent findings of improvement in ejection fraction among patients with heart failure.^21^

The benefits of SGLT2 inhibition occur in different contexts. Some benefits depend on glucosuria or the interaction between the SGLT2 inhibitor drug and the SGLT2 transporter.^14,22,23^ Increased glucosuria promotes a glucose deficit, lowers glucose and insulin in circulation, disinhibits lipolysis, increases circulating free fatty acids (FFAs) and ketone bodies, and promotes a switch in substrate utilization.^22^ Selective inhibition of SGLT2-mediated glucose and sodium reabsorption in the proximal tubule of the kidneys, leads to the complement of glucosuria, natriuresis, and osmotic diuresis. These changes promote a reduction in intravascular volume, arterial blood pressure, cardiac preload, and cardiac afterload.^2,24^ Among subjects with the triad of type 2 diabetes, chronic kidney disease, and no known preexisting ASCVD, those who had the highest glucosuria while treated with the SGLT2 inhibitor canagliflozin, also had the strongest protection against multiple cardiorenal outcomes.^22^

SGLT2 inhibitors also have effects that are independent of glucosuria. The finding that SGLT2 inhibition still confers cardiac protection in patients with low glomerular filtration rate and relatively low glucosuria and the findings in experimental models with isolated cardiomyocytes suggest that this drug class also has a direct effect on the heart.^5,11,14,23^ A study with molecular docking analysis with the SGLT2 inhibitor empagliflozin showed that empagliflozin directly binds three glucose transporters.^14^ Empagliflozin had a higher affinity for glucose transporter 1 (GLUT1) and GLUT4 compared with SGLT1. In addition, the SGLT2 inhibitor canagliflozin directly binds mammalian target of rapamycin (mTOR) in cardiomyocytes and inhibits mTOR phosphorylation.^23^ It is not clear if the mechanisms of protection are related to kidney-related systemic alterations, direct cardiovascular effects, or both.^25^

Targeted metabolomics of plasma samples in a placebo-controlled trial of dapagliflozin in patients with heart failure with reduced ejection fraction supported a role for altered ketone and fatty acid biology with SGLT2 inhibitors.^26^ This study showed that dapagliflozin increased ketone body and FFA-related metabolites, which suggested augmented consumption of both ketone bodies and FFAs in the myocardium by SGLT2 inhibitors.^27^ However, no change in cardiac energetics or targeted circulating metabolites associated with energy metabolism were found in a mechanistic cardiac MR study.^28^ This mechanistic study was designed to investigate the effects of treatment with empagliflozin on cardiac physiology and metabolism in patients with heart failure.^28^

## Lactation: Glucose Transport and Maternal Metabolism

Lactation is one of the most metabolically challenging events in the lifetime of a woman when the body prioritizes glucose transport to the mammary gland over glucose transport to most other tissues in the body.^29^ Lactation is thought to rely on the facilitated GLUT1 to transport 50–75 grams of glucose per day into the mammary epithelial cells (MECs) for the production of milk components including lactose and other carbohydrates, lipids, and proteins.^30–33^ The concentration of free glucose in milk is very low and resembles the concentration of free glucose in the cytoplasm of MECs.^34,35^ The amount of glucose transported into milk varies by blood glucose concentrations and by the intensity of the lactation stimulus (proportion of milk fed to the infant as mother’s milk relative to formula).^32,35^

GLUT1 is responsible for basal glucose transport without the mediation of insulin. Besides its ubiquitous location in adults, GLUT1 is the dominant glucose transporter in cardiomyocytes, skeletal myocytes, and liver during fetal life.^36,37^ During lactation and weaning, the proportion of GLUT1 in the tissues of the offspring decreases relative to other facilitated glucose transporters.^36^ This suggests the possibility that reliance of maternal tissues on GLUT1 during lactation resets maternal glucose uptake to an earlier program, adaptive to the uniqueness of the postpartum period. GLUT1 can transport glucose and galactose, the two monosaccharides needed to make the disaccharide lactose.^38,39^

At least three different membrane compartments participate in the transport of glucose in MECs: (1) the basolateral membrane, which is the side of the plasma membrane more proximal to maternal circulation; (2) the Golgi apparatus where synthesis of lactose takes place; and (3) the apical membrane, which is the side of the plasma membrane closest to milk.^38^ Most of what is known about glucose transport in MECs comes from studies with animals and the level of detail addressed in animal studies is impossible to attain with human studies. GLUT1 is the predominant (but not exclusive) glucose transporter in the basolateral membrane of MECs in rats, mice, and cows.^38,40^ A steep glucose concentration gradient drives glucose transport across the basolateral membranes of MECs.

In humans, the expression of a variety of glucose transporters has been identified in MECs or milk fat globules obtained from milk samples.^39,41–43^ Obermeier et al. isolated cells from fresh human milk at or after 1 month postpartum.^39^ These investigators detected SGLT1 mRNA and protein expression without indication of GLUT1 mRNA expression. SGLT1 like GLUT1 can transport glucose and galactose.^39^ This study was not designed to determine the membrane compartment(s) where SGLT1 localizes. One interpretation is that SGLT1 transport is more relevant at the apical membrane because the glucose concentration inside the MECs resembles the glucose concentration in milk. SGLT2 and GLUT4 (GLUT4 is the transporter tasked with insulin-mediated glucose uptake) have not been detected in MECs. Glucose transporters other than GLUT1/GLUT4 and SGLT1/SGLT2 have been detected by single-cell RNA sequencing of human milk-derived cell populations during early lactation and by studies on mammary cell RNA from human milk fat globules.^41,42^

In lactating women, increasing the glucose concentration in plasma resulted in a two- to three-fold increase in the glucose concentration in milk, consistent with facilitated diffusion of glucose across the basolateral membrane.^32^ Serum insulin concentrations between 2 and 70 µU/mL have no effect on human milk glucose.^32^ This glucose uptake independent of insulin concentrations is consistent with the lack of GLUT4 in MECs.^43,44^ GLUT1 and GLUT4 are the main glucose transporters in skeletal muscle and adipose tissue, the two main tissues controlling whole-body insulin sensitivity.^45–47^

The body of evidence supports that multiple glucose transporters contribute to moving glucose across the plasma membrane and organelle compartments of the MECs.^38–42^ In humans, the functional aspects of glucose transport are consistent with the predominance of GLUT1 transport in the basolateral membrane.^32^ Other glucose transporters likely contribute to the movement of glucose across MECs but in less proportion. Once glucose crosses the basolateral membrane, it is lost to maternal circulation and the interstitial space.^38^

Lastly, lactation reduces fasting glucose and insulin concentrations, characteristics that are associated with health and longevity in other contexts.^10,48,49^ Low insulin could promote nutrient deprivation signaling and autophagic flux. Lactation increases EGP in the fasting state and may reduce visceral fat, lipotoxicity, and hepatic steatosis.^50,51^ The protective association persists after adjusting for changes in body weight.

## Lactation: Mechanisms of Protection

In humans, little is known about metabolic adaptations during active lactation and postlactation responsible for about 50% reduction in the long-term risk of type 2 diabetes and metabolic syndrome in the mother.^30,52,53^ Viewing the lactating mammary gland as a sink for glucose and calories provides the simplest explanation for the immediate health benefits of active lactation on maternal metabolism. However, the leap from active benefits to lasting protection does not have a clear explanation yet.

Observational studies and studies with mice support the reset hypothesis, which states that lactation promotes recovery from the increased cardiometabolic stresses of normal pregnancy closer to the prepregnancy state.^54–56^ These pregnancy stresses include accumulation of visceral fat, hyperlipidemia, and decreased insulin sensitivity.^57,58^ The variable duration and intensity of lactation after each pregnancy result in a cumulative effect over cycles of pregnancy-lactation throughout the reproductive years. A longer lifetime duration of lactation is associated with a reduction of several maternal cardiovascular risk factors including type 2 diabetes, hypertension, hyperlipidemia, obesity, and metabolic syndrome after adjusting for other healthy behaviors.^30,53,59^ In addition, a longer duration of lactation appears to benefit the coagulation profile, particularly among women with a history of gestational diabetes.^60^ Slowing down the atherosclerosis process would likely require years of sustained improvement in risk factors for ASCVD.^1^ Importantly, the beneficial effect of lactation on cardiometabolic risk can persist for 15 years after the last birth.^30,59^

In this regard, the Coronary Artery Risk Development in Young Adults study showed that longer duration of lactation was associated with both a lower incidence of the metabolic syndrome and type 2 diabetes among parous women with and without a history of gestational diabetes, years after cessation of lactation.^30,53^ This association was present even after adjustment for weight change in the follow-up period. Lactation also reduces the risk of subclinical and clinical cardiovascular disease.^61–64^ Several studies have shown an association between lactation and improvements in the vascular health of the carotids, coronaries, and aorta.^61,63^

Among parous women in the Nurses’ Health Study, a lifetime duration of lactation of 2 years or longer was associated with a decreased risk of incident myocardial infarction compared with women who had never lactated.^64^ In addition, a recent meta-analysis that included eight observational studies and over a million pregnancies documented that any duration of lactation was associated with an 11%−17% reduction in the relative risk of cardiovascular disease, coronary heart disease, stroke, and fatal cardiovascular disease.^65^ To date, there are no published clinical studies on the effect of lactation on the prevention of heart failure. However, translational work with mice has shown increased ejection fraction as a benefit of lactation versus no lactation.^55,56^ This increase in ejection fraction was already present at 2 months postpartum (after weaning) and extended into the mouse midlife at 9 months postpartum.

## Effects of SGLT2 Inhibition and Lactation on Weight and Body Composition

SGLT2 inhibition and lactation share several characteristics that result in changes in body weight and composition. These changes include loss of energy in a body fluid (urine or milk) and a compensatory increase in caloric intake that result in lower-than-predicted weight loss.^66–68^ Both SGLT2 inhibition and lactation decrease waist circumference, percent body fat, visceral, subcutaneous, and hepatic fat.^50,51,69–72^ However, their effect on lean mass and skeletal muscle mass is variable.^69,73^

The glucosuria induced by SGLT2 inhibitors (50–100 grams/day) has an equivalent loss of energy in the urine of 200–400 kcal/day.^7^ This energy loss triggers an increase in caloric intake that limits weight loss to 2–4 kg.^7^ Two-thirds of the weight loss is fat mass and one-third is fat-free mass.^66^ SGLT2 inhibitors induce weight loss in patients with and without diabetes and decrease fat in soft tissue compartments: visceral, subcutaneous, hepatic, and epicardial.^70,71,74,75^ This drug class also decreases lean and skeletal muscle mass.^69^

The trajectories of weight and body composition during lactation are influenced by the trajectories of weight and body composition during pregnancy.^58^ Weight gain during pregnancy impacts the patterns of postpartum weight retention between lactating and nonlactating women partly because weight gain during pregnancy is the major determinant of postpartum weight retention.^76^ Although the direction and magnitude of weight change during lactation varies, women who lactate during the first year postpartum lose on average 2 kg more than formula-feeding women from 1 to 12 months postpartum.^67^ Women also experience an increase in visceral fat during pregnancy.^58^ Longer duration of lactation is associated with lower visceral fat volumes before and after menopause as well as a lower risk of metabolic dysfunction-associated steatotic liver disease in midlife.^50,51,77^

## Differences Between SGLT2 Inhibition and Lactation

SGLT2 inhibitors and lactation have important differences regarding the onset and duration of alterations in metabolism, target organs that reap protective effects, the magnitude of the starvation stimulus, and the effect on insulin secretion and insulin sensitivity. The benefit obtained during active treatment with SGLT2 inhibitors appears to cease with discontinuation of therapy. Treatment with SGLT2 inhibitors induces an increase in glucagon concentration and fat oxidation as well as the development of ketosis in a sort of accelerated starvation.^6^ SGLT2 inhibitors improve insulin secretion when adjusting for glucose during a hyperglycemic clamp.^8^ SGLT2 inhibitors improve insulin sensitivity during a hyperinsulinemic-euglycemic clamp after correction for urinary loss of glucose.^9^

The pharmacologic inhibition of SGLT2 in the proximal renal tubule and the stimulation of nutrient transport into the lactating mammary gland result in loss of calories in their respective body fluids, urine, or milk. The amount of glucose excreted in urine in response to SGLT2 inhibition increases in proportion to the plasma glucose concentration.^5^ In contrast, women who only feed their infants with mother’s milk produce an average 750–800 mL of milk per day once lactation is established.^31,34,78,79^ Considering an energy content of 65–70 kcal per 100 mL of milk, the amount of energy in milk ranges from 480 to 560 kcal/day with a mixed macronutrient composition that includes fat, carbohydrate, and protein.^80^ About 55% of this energy is fat, 40% is carbohydrate, and 5% is protein.^32,81,82^

Lactation is associated with cardiometabolic benefits long after weaning.^30,53^ Compared with women who only feed formula, lactating women have lower glucagon concentrations at 3 and 6 months postpartum, suppression of lipolysis at lower insulin concentrations, and onset of ketosis following a 24–42-hr fast rather than during an overnight fast.^31,49,83^ These characteristics suggest that compared with SGLT2 inhibition, the starvation stimulus during lactation is gentler on nonmammary tissues. Although lactating women exhibit similar or decreased insulin secretion during a 2-hr oral glucose tolerance test (OGTT) or during an intravenous glucose tolerance test (IVGTT) when compared with formula-feeding women, lactating women also exhibit an improved disposition index during an IVGTT.^84–88^ These changes suggest that the insulin secretory response is appropriate during lactation.

**FIG. 1.**
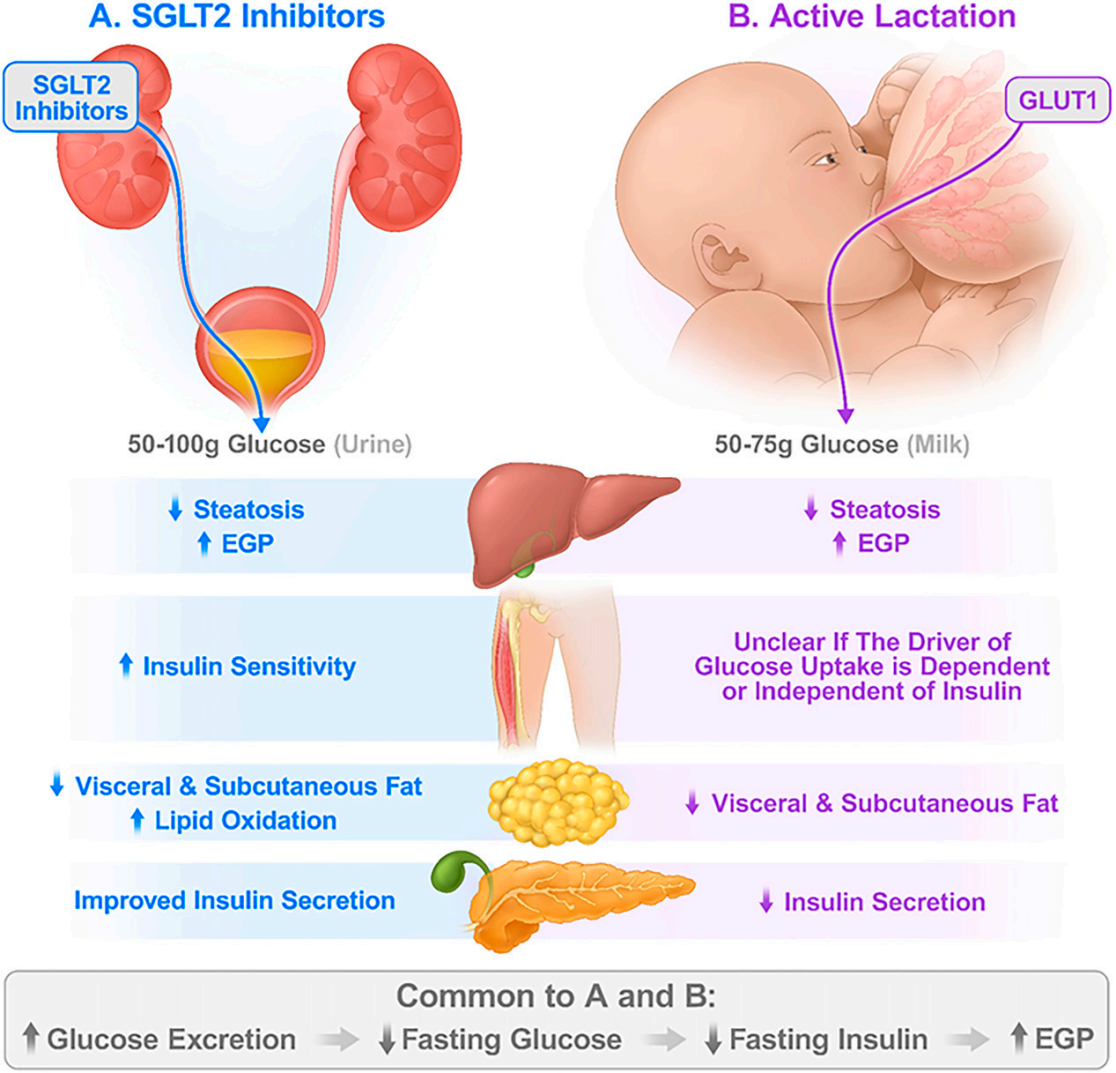
Effect of SGLT2 inhibitors and lactation on the main tissues involved in glucose metabolism. **(A)** SGLT2 inhibitors induce excretion of 50–100 grams of glucose per day in urine. This drug class promotes a systemic response that mimics starvation despite also inducing hyperphagia. The responses in major metabolic tissues include decreases in hepatic steatosis, visceral and subcutaneous fat, and a mean weight loss of 2–4 kg; and increases in endogenous glucose production (EGP), lipid oxidation, and insulin sensitivity. Insulin secretion improves during hyperglycemic clamps. **(B)** Active lactation induces excretion of 50–75 grams of glucose per day in milk and is associated with hyperphagia. The responses in major metabolic tissues include decreases in hepatic steatosis, visceral and subcutaneous fat, and increases in EGP. It is unclear how insulin-sensitive are the nonmammary tissues of lactating women. It is not known if glucose uptake in nonmammary tissues is mainly dependent or mainly independent of insulin. Relative to formula-feeding women, insulin secretion decreases during lactation while the amount of secreted insulin for the magnitude of insulin resistance increases.

Specific to insulin sensitivity, longitudinal assessments between pregnancy and 3 years postpartum using OGTT showed better insulin sensitivity by Matsuda Index in women who lactated 12 months or longer compared with women who lactated 3 months or less and in women with a history of lactation versus no lactation.^84,89^ Published work with hyperinsulinemic-euglycemic clamps during lactation has not accounted for the loss of glucose into milk during calculations of insulin-stimulated glucose disposal.^35,85^ A large proportion of glucose uptake during lactation appears to be noninsulin-mediated glucose uptake, typically associated with GLUT1 transport. Therefore, adaptations in glucose transport may confound the interpretation of whole-body and tissue-specific sensitivity to insulin among lactating women. Figure 1 compares the important effects of SGLT2 inhibitors and lactation on metabolism.

## Differential Effects of SGLT2 Inhibition and Lactation on Glucagon and Incretin Hormones

Glucagon potentiates glucose-stimulated insulin secretion (GSIS), stimulates energy expenditure and hepatic glucose production (HGP), and decreases glucose effectiveness.^90–93^ Glucose effectiveness refers to the ability of glucose itself to suppress EGP and stimulate peripheral glucose uptake as noninsulin-mediated glucose uptake.^93^ Glucagon-like peptide 1 (GLP-1) potentiates GSIS, induces satiety, decreases food intake, inhibits HGP, and increases glucose effectiveness.^91,93–95^ Glucose-dependent insulinotropic polypeptide also potentiates GSIS and increases glucose effectiveness.^93,95^

Specific to SGLT2 inhibition, the alpha cells in human islets express *SLC5A2* mRNA (encoding SGLT2).^96^ The expression of *SLC5A2* as well as glucagon secretion exhibit marked interindividual variation, which may contribute to the range of glucagon responses to SGLT2 inhibitors in patients with diabetes.^96^ The metabolic effects of SGLT2 inhibitors vary along the spectrum of glucose tolerance.^6^ In the context of normal glucose tolerance (NGT), treatment with SGLT2 inhibitors did not affect fasting plasma glucose, insulin, or glucagon concentrations despite glucosuria of about 50 grams/day.^6,97^ Individuals with NGT had stable mean fasting plasma glucagon concentrations ranging from 69 to 75 pg/mL during the 14-day period of study before and after the introduction of SGLT2 inhibition. Furthermore, adults with obesity and without diabetes had similar plasma glucagon when treated with SGLT2 inhibitors or placebo.^71^ The intermediate group with impaired fasting glucose had a small but significant decrease in fasting plasma glucose with no change in fasting plasma insulin or glucagon.^6^ Lastly, adults with type 2 diabetes had a significant decrease in fasting plasma glucose and a significant increase in fasting plasma glucagon within 1 day of the introduction of SGLT2 inhibition.^6^

Two studies comparing fasting plasma glucagon in lactating versus nonpregnant, nonlactating women reported similar glucagon concentrations between the groups (∼76 pg/mL).^31,98^ A longitudinal evaluation of well-nourished women followed between term pregnancy and 6 months postpartum, reported higher mean glucagon concentrations during pregnancy (∼160 pg/mL) compared with the postpartum period and higher serum glucagon in nonlactating (∼138 pg/mL) compared with lactating women at 3 months (81 pg/mL) and at 6 months postpartum (83 pg/mL).^49^ These three independent studies reported similar fasting glucagon concentrations among lactating women.^31,49,98^ A fourth study on this subject reported fasting and postprandial concentrations of GLP-1 at 4–6 weeks postpartum in women who only fed their infants with mother’s milk versus a never-pregnant control group. This study found no differences in GLP-1 concentrations between the groups, which were matched for age and body composition.^99^

Lactation is characterized by low insulin concentrations, increased EGP in the fasting state, increased appetite, and presumed increased glucose effectiveness because of the reliance of a large proportion of glucose utilization on noninsulin-mediated glucose transport to the MECs for milk production. The contribution of the vast concurrent and sequential hormonal changes that occur during pregnancy and the postpartum period to these processes once lactation is established is not clear.

## Implications for Cardiometabolic Health in Parous Women

SGLT2 inhibition and lactation result in the excretion of large amounts of glucose in urine or milk and are associated with a lower risk of cardiovascular events. The mechanisms behind the association of SGLT2 inhibition with cardiovascular protection are not clear. In whole organisms, it is difficult to separate the systemic contribution of glucosuria from direct cardiovascular effects when SGLT2 inhibitors bind to molecules other than the SGLT2 transporter, which is not expressed in cardiomyocytes. Likewise, the mechanisms behind the association of lactation with cardiovascular protection are not clear. The reset hypothesis links relatively small but sustained improvements in a cluster of cardiometabolic risk factors (blood pressure, lipids, glycemia, body weight, and body composition) with fewer cardiovascular events.^54^ The reset hypothesis is a compelling argument when considering that slowing down the atherosclerosis process would likely require years of sustained improvement in its risk factors.^1^

In general terms, the systemic effects observed with SGLT2 inhibitors and lactation result from the combination of tissue-specific effects and inter-tissue communication. SGLT2 inhibition and lactation involve glucose transport. SGLT2 inhibition and lactation also affect cardiometabolic risk. Despite similarities in many metabolic effects, their specific mechanisms of protection may differ. Other potential mechanisms for improvement in metabolic health among parous women are lactation and postlactation effects on bone health and stem cells.^100^ The duration of postlactation recovery can be defined as the 6 to 12 months that it takes the maternal skeleton to recover from the bone loss induced by lactation.^101^ Such recovery implies prolonged changes in the bioenergetics of bone to build back to the prepregnancy state.

In terms of stem cells, Avogaro et al. suggested that the energy deficit with SGLT2 inhibitors induces a dormancy program linked to autophagy and stem cell quiescence.^17^ Skeletal stem and progenitor cells (SSPC) are known to rejuvenate in contexts other than the pregnancy-postpartum periods under conditions of energy deficit that upregulate autophagy.^102–104^ Avogaro et al. asked, “Can the effect of SGLT2 inhibitors on cellular reprogramming occur with other noninsulin-dependent reductions in plasma glucose concentration?”^17^ If cellular reprogramming occurred during lactation, would it be possible for the rejuvenation of SSPC to partly explain the durable cardiometabolic benefit associated with lactation? Learning from the similarities and differences between SGLT2 inhibitors and lactation will advance our understanding of cardiometabolic health for all people. Clinical research on the physiology of lactation and the postlactation period is critically needed to promote the health of parous women during their reproductive years. Only then will the mechanisms behind this protective association be identified.
